# A Unique Case of Low-Grade Mucinous Neoplasm in Stump Appendectomy

**DOI:** 10.1155/2020/8850403

**Published:** 2020-09-14

**Authors:** Mohannad Al-Tarakji, Syed Muhammad Ali, Amjad Ali Shah, Mahir Abdulla Petkar, Salman Mirza, Rajvir Singh, Ahmad Zarour

**Affiliations:** ^1^Department of Acute Care Surgery, Hamad Medical Corporation, Doha, Qatar; ^2^Department of Pathology, Hamad Medical Corporation, Doha, Qatar; ^3^Department of Radiology, Hamad Medical Corporation, Doha, Qatar

## Abstract

**Background:**

We describe a case of a young male with a history of appendectomy one year ago, who developed symptoms of stump appendicitis, and after removing this stump, histopathology showed low grade neoplasm. *Summary*. Stump appendicitis is an uncommon complication after appendectomy and may lead to serious complications. Management of low-grade appendiceal mucinous neoplasm (LAMN) is controversial, and we discuss the importance of the case.

**Conclusion:**

The case of young male post stump appendectomy with histopathology showing LAMN in the stump of the appendix, which to our knowledge, is the first in the medical literature and, discuss the stump appendicitis and incomplete appendectomy concerning malignancy, mucinous neoplasm, and adenocarcinoma.

## 1. Introduction

Stump appendicitis, the remnant of appendiceal tissue after surgical removal of an inflamed organ, is one of the rare complications after appendectomy [1] whether performed open or laparoscopically. The time of presentation is variable, and the patient can develop almost identical symptoms of acute appendicitis. Computed tomography (CT) scan makes the diagnosis or, subsequently, by exploration. Treatment can range from antibiotics to surgery. Further treatment, if needed, after stump appendectomy depends on histopathology findings.

## 2. Case Description

**Figure 1:**
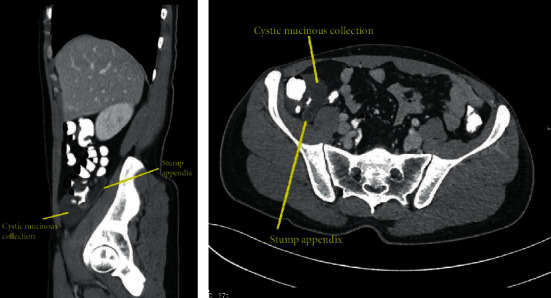
These sagittal and axial cuts from CT scan after appendectomy shows fluid-filled mildly distended appendicular stump with no abnormally thickened wall. An adjacent well-defined fluid collection is seen caudally, showing a density of 20 Hounsfield units.

**Figure 2:**
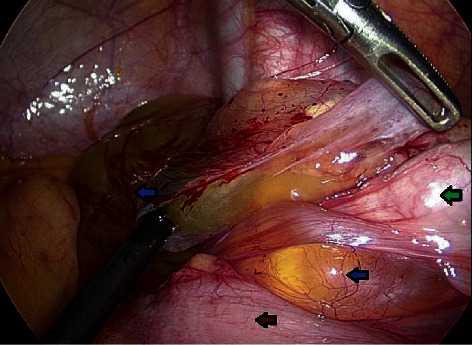
Operative photograph of laparoscopic exploration showing cecum (green arrow) gelatinous material (blue arrows) and terminal ileum (red arrow).

**Figure 3:**
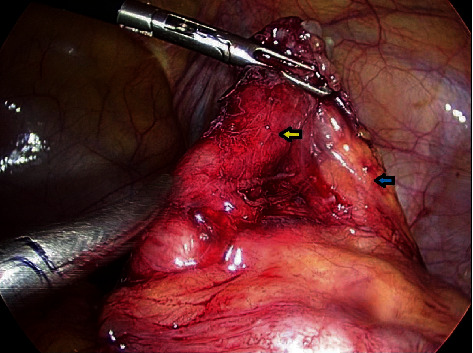
Operative picture of the stump (yellow arrow) and mesentery (blue arrow) after the gelatinous material has been separated. The tip was attached to the mucoid jelly-like material and shows abnormal tissue.

**Figure 4:**
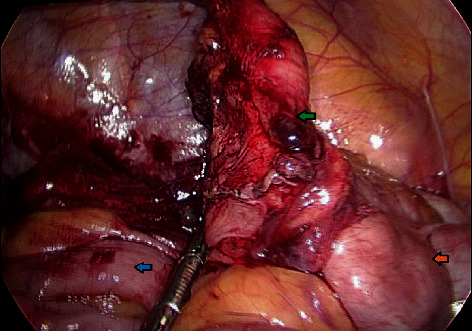
Laparoscopic view of the stump (green arrow), cecum (orange arrow), and terminal ileum (blue arrow).

A 36-year-old male with no chronic disease admitted for laparoscopic exploration for intermittent abdominal pain after an open appendectomy that was performed 11 months ago. On previous admission, he was operated for clinical and radiological diagnosis of acute appendicitis. Initial CT scan showed distended appendix measuring 8-9 mm in diameter, showing abnormally thickened enhancing the wall, with surrounding fat stranding, free fluid, and adjacent pocket of pus collection measuring 2 × 4 cm. Open appendectomy was performed with findings of the gangrenous appendix, where the base was healthy and appropriately secured with Vicryl suture. He was discharged in good condition two days postsurgery. Pathology of the specimen showed 4.5 cm long appendix and 0.7 cm in diameter with a diagnosis of acute appendicitis and periappendicitis. After almost a year, he was seen in the clinic as he complained of recurrent abdominal pain for one month and a slight decrease in appetite. His ultrasound abdomen showed a complex heterogeneous collection with internal echoes in the right iliac fossa measuring 5.5 × 2.7 × 6.4 cm, corresponding to a volume of 50 ml. His CT abdomen showed a well-defined mass-like lesion about 5 × 5 cm in diameter of fluid density (22 HU) suggesting thick fluid in the right iliac fossa; the stump of the removed appendix is well demonstrated and about 4 cm in length and closely related to the lower part of this collection (Figure 1). The patient underwent laparoscopic exploration with excision of the subserosal appendicular stump of about 5 cm and cyst extending from the tip and distal part of the appendix to the pelvis, easily separable from the pelvis, containing gelatinous yellowish material (Figures 2–4). The terminal ileum and right-sided colon were normal, and no peritoneal deposits were observed. Because of these findings, we resorted only to stump excision.

**Figure 5:**
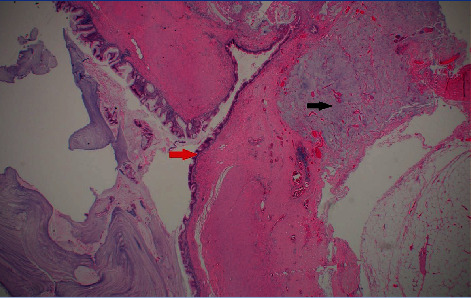
Low-power view of the lesion, H and E ×10. Red arrow highlights the dysplastic mucosa. Note the mucin in the wall (black arrow).

**Figure 6:**
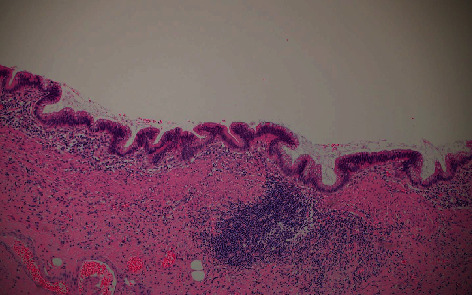
High power displaying the atypical mucosa, H and E ×20.

**Figure 7:**
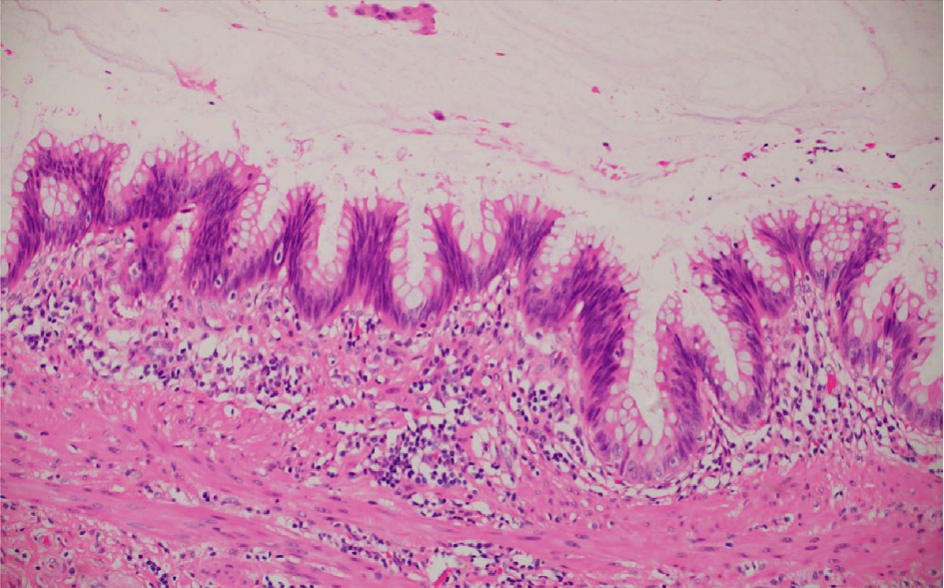
Dysplastic mucosa lining the lesion, H and E ×40.

Cytology of the gelatinous fluid was negative for malignant cells. At the same time, the histopathology of the stump showed appendix measuring 4 cm in length and 0.6 cm in diameter with low-grade appendiceal mucinous neoplasm (LAMN) (Figures 5–7). The tumor is located in the distal half of the appendix, with a size of 0.6 cm in the greatest dimension. The base of the appendix was uninvolved by a tumor with a distance of 1 cm, while acellular mucin invaded subserosa but did not extend to the serosal surface or proximal margin. No lymph nodes were identified in the specimen. The pathologic classification was pT3 pNX (pTNM, AJCC Eighth edition). The previous specimen of the appendix was reexamined, wherein, no evidence of appendiceal mucinous neoplasm was found. CT thorax and abdomen with contrast, as part of staging, showed no evidence of metastatic disease, lymphadenopathy, or active inflammatory process. Colonoscopy was normal up to the cecum and terminal ileum. CEA and CA19-9 were 2 and 5.1 units/L, respectively. The case was discussed in gastrointestinal cancer multidisciplinary team (MDT) with a recommendation of close follow-up as the patient had a high risk for developing pseudomyxoma peritonei. The last follow-up was three months ago, and he was symptom-free.

## 3. Discussion

Stump appendicitis is the inflammation of the residual appendiceal tissue after an appendectomy. It is a rare complication with a frequency that is underreported as well as underestimated. Its incidence was reported to be 1 in 50,000 cases [1]. Rose reported the first case of stump appendicitis (SA) in 1945 [2]. Most of the complications of appendectomy include wound infection, periappendicular or intra-abdominal abscess, and postoperative adhesions [3]. The other late technical complication is stump appendicitis [4–8]. There is no consensus regarding how to avoid stump appendicitis. In the literature, causes of stump appendicitis are an insufficient inversion of a stump, a remnant of excessive length, and insufficient laparoscopic appendectomy [3–6].

Appendiceal malignancies are uncommon and include carcinoids, cystadenocarcinoma, and adenocarcinomas [9]. They usually have no specific clinical signs, symptoms, or radiologic features [10, 11], and preoperative diagnosis is, therefore, tough [12]. The diagnosis is most often made after appendectomy or at autopsy. The first case of carcinoma of the appendix was described by Berger in 1882 [13]. It constitutes 0.2%-1.0% of all intestinal malignant tumors [14], while the incidence of adenocarcinoma in appendectomy specimens has been reported as ranging from 1/700 to 1/1600 [15]. Collins, in another study, found that adenocarcinoma of the appendix has been found in only 0.08% of appendices removed for disease, incidentally or at autopsy, in a sample size of 71000 [16]. Carcinoid of the appendix occurs ten times more common than primary adenocarcinoma [17]. Appendiceal mucinous neoplasms account for 0.4%–1% of all gastrointestinal malignancies in the U.S., roughly translating to 1,500 new cases annually [16, 18–21].

LAMNs is low-grade and shows uncertain malignant potential. It is a well-differentiated adenoma that can proliferate outside the appendix in malignant fashion [22]. LAMN confined to the mucosa are classified as adenomas and those that display pushing or expansile invasion and can disseminate in the peritoneal cavity as pseudomyxoma peritonei (PMP), for which the term low-grade appendiceal mucinous neoplasm (LAMN) was proposed [23] and adopted by the World Health Organization [22]. The usual age of LAMN at presentation was near 56.7 years, but in our case, it was in a young male of 36, conforming to previous reports [23, 24]. There might be elevated levels of CEA, CA 19-9, and CA-125 may be detected in 56.1-67.1% of patients [25]. There is a 35% risk of a concurrent GI malignancy in patients with LAMN [26], and therefore, these patients need follow-up.

The goal of management of LAMN includes the prevention of rupture, seeding, and development of pseudomyxoma peritonei (PMP) [27]. Low-grade appendiceal mucinous neoplasms with acellular or cellular mucin in the right lower quadrant are at low and high risk, respectively, for dissemination in the peritoneal cavity. That risk is not modified by the status of the surgical resection margin [28, 29]. However, low-grade appendiceal mucinous tumors infrequently involve lymph nodes, even when they have spread to the peritoneum. Thus, the role of right hemicolectomy in patients with the disseminated peritoneal disease (i.e., pseudomyxoma peritonei) is not clear. Some studies have shown that right hemicolectomy can have a negative survival advantage in the setting of pseudomyxoma peritonei and should only be carried out at the time of complete cytoreduction and intraperitoneal chemotherapy, as the success of the latter may be impaired by adhesions created by prior colonic surgery [30, 31]. The involvement of the appendectomy margin by either neoplastic epithelium or acellular mucin is not associated with disease recurrence or peritoneal dissemination [32]. Surveillance of patients with LAMN incorporates radiographic imaging every six months for two years after appendectomy for adequate monitoring of tumor recurrence and complications associated with PMP [33]. While other articles suggested, follow-up should continue for five to 10 years with physical exams, annual CT, and monitoring of tumor markers. The five-year survival rate for localized LAMN is 95% [34].

As far as the appendiceal stump carcinoma is concerned, it was first reported by de Ruyter in 1903 at autopsy, in which a carcinoma was found in the stump of an appendix. The appendix was removed six years back [35]. This was followed by another report by Gamble of a patient who was found to have colonic intussusception due to adenocarcinoma of appendiceal stump 43 years after appendectomy [36]. Our patient is unique as it reports the first LMAN in stump appendicitis in a young male, which is not the usual age of presentation. As reviewed, the management of LAMN still controversial; it needs more data and follow-up to clarify the role of close follow-up versus surgery to eliminate the risk of pseudomyxoma peritonei and malignant transformation.

## 4. Conclusion

Low-grade mucinous neoplasm is very rare in stump appendectomy with no reported cases previously. Treatment varies based on the cellularity of mucin ranging from hemicolectomy to close follow-up as advised in case of noncellular mucin such as in our case. Further studies and prolonged follow-up will add to the management of LAMN. Patient education about the case and the follow-up plan is crucial in high-risk patients for pseudomyxoma peritonei.

## Data Availability

The data used to support the findings of this study are included within the article.

## Additional Points

*Lessons Learned*. Stump appendicitis despite being an uncommon surgical disease postappendectomy, it might be hiding a malignant potential. Management of any appendiceal malignancy should follow the up to date guidelines specific to each pathology.
